# Defect induced improved capacitive performance of MnS incorporated MoO_3_ nanocomposite for supercapacitor electrodes in aqueous electrolytes

**DOI:** 10.1371/journal.pone.0349019

**Published:** 2026-05-18

**Authors:** Mizanur Rahaman, Mehedi Hasan Prince, Saif Mahmud Bijoy, Zakaria Siddiquee, Muhammad Rakibul Islam

**Affiliations:** 1 Department of Physics, Bangladesh University of Engineering and Technology, Dhaka, Bangladesh; 2 Department of Physics, Kent State University, Kent, Ohio, United States of America; 3 Department of Materials and Metallurgical Engineering, Bangladesh University of Engineering and Technology‌‌, Dhaka, Bangladesh‌‌; 4 Advanced Materials and Liquid Crystal Institute, Kent State University, Kent, Ohio, United States of America; Universidad Autónoma de Querétaro: Universidad Autonoma de Queretaro, MEXICO

## Abstract

Electrode materials play a crucial role in improving supercapacitor performance. In this work, MnS nanoparticles were incorporated into MoO_3_ to form a MoO_3_/MnS nanocomposite via hydrothermal synthesis, and the capacitive performance of the resulting supercapacitor electrodes was evaluated. Their electrochemical performances were studied in conjunction with KCl and Na_2_SO_4_ electrolytes. The generation of MoO_3_/MnS nanocomposite was confirmed by XRD analysis and HR-TEM imaging. It is found that the MnS nanoparticles altered the morphology of MoO_3_ from nanobelts to nanofibers and produced a defective, rough surface. The defective surface expanded the interlayer distance from 0.396 nm to 0.421 nm. In both ionic electrolytes, the MoO_3_/MnS composite demonstrated higher capacitive performance than the pristine MoO_3_. At 0.3 A g^-1^ current density, the estimated specific capacitance of MoO_3_/MnS was 387 F g^-1^ and 335 F g^-1^ in KCl and Na_2_SO_4_ electrolytes, respectively. In the symmetric two-electrode system, the MoO_3_/MnS shows a specific capacitance of 297 F g^−1^ at 1 A g^−1^, with an energy density of 33.37 Wh kg^−1^ and a power density of 450 W kg^−1^. The MoO_3_/MnS nanocomposite provides excellent 90% retention after 1000 continuous charging-discharging cyclic. The enhancement of electrochemical performance is attributed to the large surface area, defective morphology, and broader interlayer distance. This system bridges the gap between traditional batteries and capacitors, offering a unique approach to producing supercapacitor electrodes.

## 1 Introduction‌‌

The rapid advancement of human civilization and technology in recent times has been largely driven by the widespread use of fossil fuels. The burning of fossil fuels harms our environment and is being depleted rapidly. The use of sustainable energy, such as sunlight and wind, requires reliable energy storage. Electrical energy can be stored either electrochemically in batteries or electrostatically in capacitors. Batteries have ~50–200 Wh/kg energy density and low (~1–1000 W/kg) power density, while electrostatic capacitors have energy densities less than 0.1Wh/kg and power densities over 5000 W/kg [1]. The gap between batteries and capacitors has been partially bridged by supercapacitors, which are currently used in power conditioning and electric transportation. Supercapacitors offer notable advantages such as long cyclic life, relatively fast charge and discharge, and high-power density, which makes a bridge between batteries and capacitors [2,3]. Generally, electrode materials play a significant role in enhancing the performance of supercapacitors [4–7].

Transition-metal oxides such as molybdenum trioxide (MoO_3_) have gained significant interest as electrodes due to their high theoretical capacitance (1,005 C/g), exceptional cation accommodation efficiency, favorable charge transfer ability, and semiconducting properties [8–10]. MoO_3_ has three different polymorphs. Among them, α-MoO_3_ is remarkably important due to its structural anisotropy and the alternating stack of MoO_6_ octahedra double layer bound by the Van Der Waals force along the [001] direction [11]. However, MoO_3_ is prone to structural instability, has poor electrical conductivity, and limited rate capacity that reduces the electrode’s electrochemical properties for supercapacitor applications [12]. The electrochemical performance of MoO_3_ can be improved by enhancing its electrical conductivity, surface area, and interlayer spacing.

The incorporation of nanostructured materials is an efficient approach for improving the capacitance of the oxide-based materials. The incorporation enhances surface area, improves conductivity, expands interlayer distance, and generates electrochemically active sites — all of which help achieve better capacitive performance. [4] Several studies have been performed on the supercapacitor applications of MoO_3_-based nanocomposites. Zhou et al. fabricated an Ag-decorated MoO_3_ nanocomposite for a supercapacitor electrode in a liquid phase method [13]. They achieved the highest specific capacitance of 225 F g^-1^ and 71.1% cyclic stability with 8% Ag content in the Ag@MoO_3_ nanocomposite. Capacitive performance and cyclic stability may not fully reflect the stability of a supercapacitor electrode. Imran et al. used the hydrothermal method to produce intertwined porous MoO_3_–MWCNT nanocomposites [14]. The measured specific capacitance is 210 F g^−1^at the lowest scan rate, 5 mV s^−1^. At the lowest scan rate, ion diffusion is very slow, resulting in a longer time to complete the full cycle. Sadananda et al. grew ZnO nanoparticles on MoO_3_ via the solid-state impregnation-calcination method [15]. This nanocomposite is combined with carbon black, yielding a specific capacitance of 280 F g^–1^ at a current density of 1 A g^–1^. In this work, the carbon black modifies the nanocomposites’ original capacitance.

Recently, MnS nanoparticles gained considerable attention due to their high theoretical capacitance, strong redox reactions, charge transfer kinetics, and higher electronic conductivity (3.2 × 10^3^ S cm^−1^) compared to their oxide counterparts [16]. Nanostructured MnS demonstrates high ionic penetration and intercalation–deintercalation properties, contributing to the electrochemical stability of supercapacitors. Moreover, when combined with other materials, MnS shows improved performance [17]. The polymorph structure of MnS plays a vital role in improving the electrochemical performance of MoO_3_ [18]. Moreover, MoO_3_/MoS_2_ binary nanocomposites have shown significantly high specific capacitance and cycling stability [19]. Another recent study on MoO_3_/MoS_2_ nanocomposite electrode has demonstrated ultrahigh capacity and excellent rate performance, highlighting the advantages of such hybrid structures for pseudocapacitive energy storage [20]. Other research has also studied morphology and defect-controlled α- [20] MoO3 structures, showing that morphologies with higher surface area and defect sites can significantly improve charge storage capacity [21]. Beyond MoO_3_/MoS_2_ systems, various metal sulfide-modified MoO_3_ composites, including bimetallic sulfide/oxide heterostructures, have been shown to enhance electrical conductivity and charge transport, providing a promising strategy to overcome the inherently low conductivity of pure MoO_3_ [22]. Although reports on MoO3/MnS nanocomposites specifically for supercapacitors are limited, results from related metal sulfide and MoO3/metal sulfide systems suggest that forming heterostructures and introducing defects may enhance electrochemical performance. Several MoO_3_-sulfide-based systems have been explored for electrochemical energy storage, reports on MnS-incorporated MoO_3_ nanobelts synthesized via hydrothermal processing and systematic evaluations in different aqueous electrolytes are lacking. In particular, the role of MnS-induced defects in enhancing the capacitive performance of MoO_3_ has not yet been comprehensively investigated.

In this study, we used a facile hydrothermal approach to synthesize MoO_3_ nanobelts and MoO_3_/MnS nanocomposites and studied their structural, morphological, and electrochemical properties. The hydrothermal process was chosen because it produced approximately 85–90% of the final product relative to the precursor mass. Repeated batches prepared under identical conditions demonstrated consistent phase purity and electrochemical performance, indicating reliable batch-to-batch reproducibility. The incorporation of MnS drastically changed the morphology of the MoO_3_ nanobelts to nanofibers, generating defects and pores. The defective porous morphology increases the specific surface area, number of active sites, thereby enhances the nanocomposite’s electrochemical properties. The nanocomposite shows specific capacitances of 387 F g^-1^ and 335 F g^-1^ at current density 0.3 Ag^-1^ in 0.5M KCl and 0.5M Na_2_SO_4_ electrolytes, respectively. These results demonstrate remarkable enhancement of the capacitive performance of MoO_3_/MnS nanocomposite thus opening a new way for improving supercapacitor electrodes.

## 2 Experimental section

### 2.1 Synthesis of MoO_3_, MnS and MoO_3_/MnS nanocomposites

For the hydrothermal synthesis of MoO_3_ nanobelts and MnS nanoparticles, the precursors, Sodium Molybdate (Na_2_MoO_4_.2H_2_O) and Hydrochloric Acid (HCl), Manganese (II) chloride tetra-hydrate (MnCl_2_.4H_2_O), and Hydrazine hydrate (N_2_H_4_), were purchased from Merck, India.

Initially, 0.08 M Na_2_MoO_4_.2H_2_O was dissolved in 120 ml of DI water and stirred. After that, HCl (~3 ml) was added dropwise, the pH was maintained at 1, and the solution was stirred for 30 minutes to form a homogeneous solution. The mix was subsequently moved to a Teflon-lined autoclave, heated for 24 hours at 150 °C, and allowed to cool to room temperature. Following centrifugation, the yield was cleaned more than three times with ethanol and DI water to eliminate impurities. First, a specific amount of MnCl_2_· 4H_2_O and C_2_H_5_NS was combined in 120 mL of DI water, stirred for 1 hour. The past solution was merged with hydrazine hydrate (N_2_H_4_), and the mixture was agitated for 2 hours to form a homogeneous solution. The solution was then transferred to the autoclave and heated to 180 °C for 24 hours. The precipitate was washed several times with ethanol and DI water and dried at 60 °C for 3 h on a hot plate to obtain the MnS nanoparticles.

To prepare the MnS-incorporated MoO_3_ nanocomposite, first, 5 wt% MnS nanoparticles were dissolved in 50 mL DI water and sonicated for 1 hour. Then it was mixed with 70 mL of a MoO_3_ precursor solution and stirred for 30 minutes. The solution was heated in the autoclave to 150° C for 24 hours, then cooled to room temperature. Finally, the resulting MoO_3_/MnS nanocomposite was washed numerous times and dried at 60 °C.

### 2.2 Characterization

The surface morphology of MoO_3_ and MoO_3_/MnS was examined by a field emission scanning electron microscope (JSM 7600, JEOL) and a transmission electron microscope (JEM 2100 F, JEOL). For the structural analysis, X-ray diffraction (XRD) patterns of the samples were obtained by utilizing an X-ray diffractometer (PANalytical Empyrean) equipped with a Cu-Kα X-ray source (λCuKα = 1.54278 Å). The electrodes’ electrochemical behavior was tested using a CS310 (Cortest, China) workstation in standard three-electrode configurations with a modified graphite working electrode, an Ag/AgCl reference electrode, and a 1 cm × 1 cm platinum counter electrode plate. 0.5 M Na_2_SO_4_ and 0.5 M KCl aqueous ionic electrolytes were used in a 0.1V − 0.7 V voltage window. For the working electrodes, polyvinyl alcohol (C_2_H_4_O) and dimethyl sulfoxide (C_2_H_6_OS) solvents were mixed with the electrode materials. Afterwards, these mixed solutions were put uniformly (0.3 mg active mass loading) via drop casting on the surface of modified graphite electrode and dried 30 min at 60 °C.

## 3 Results and discussion

### 3.1 Morphological analysis

**Fig 1:**
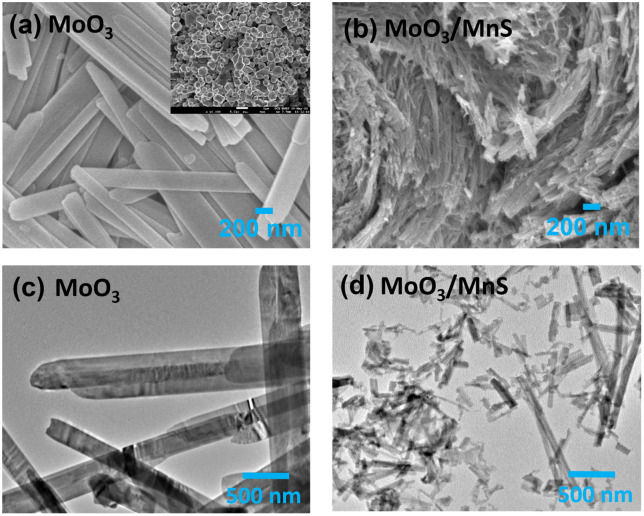
Images of MoO_3_ nanobelts and MoO_3_/MnS nanocomposites. **(a)** FE-SEM images of MoO_3_ nanobelts; **(b)** FE-SEM images of MoO_3_/MnS nanocomposites; **(c)** TEM images of MoO_3_ nanobelts; **(d)** TEM images of MoO_3_/MnS nanocomposites.

Fig 1(a) shows the FE-SEM image of MoO_3_ nanobelts formed after hydrothermal reaction with 200–300 nm diameters and 5–10 μm lengths. The inset of Fig 1 (a) shows FE-SEM image of MnS nanoparticles. The MoO_3_ nanobelts aggregate because of their large surface energy and surface tension, which minimizes their specific surface area [23]. After incorporation of MnS nanoparticles, the morphology of MoO_3_ changed from nanobelts to nanofibers, as shown in Fig 1(b). By using ImageJ software, the measured nanofibers diameter is 30–40 nm. The MnS nanoparticles shrink to the width of the MoO_3_ belts, resulting in an increase of specific surface area that improves the capacitive performance. The MnS nanoparticles are randomly distributed in the MoO_3_ medium, creating a rough surface and numerous pores in MoO_3_. Such rough and porous morphology of the MoO_3_/MnS nanocomposites significantly increases the specific surface area, therefore, supplies more electrochemically active sites, which can accommodate the volume expansion [24]. This enhances the overall capacitive performance of MnS injected MoO_3_ nanocomposites. Due to this unique morphology, the impedance of the material greatly reduces and provides rapid charge transportation, thereby enhancing capacitance [25].

Fig 1 (c, d) shows TEM micrographs of MoO_3_ nanobelts and MoO_3_/MnS nanocomposites. From TEM micrographs, it is evident that MoO_3_ forms nanobelts that connect with one another. In the case of MoO_3_/MnS nanocomposites, it is evident that the nanobelts break down, producing rich, porous nanofibers with defects.

**Fig 2:**
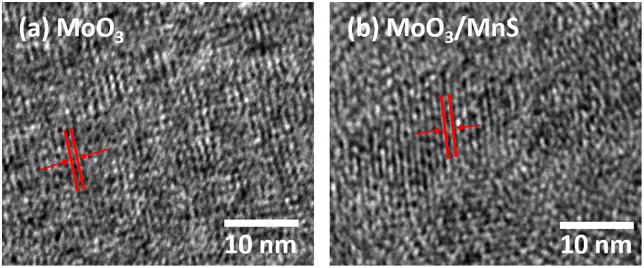
HR-TEM images of (a) pristine MoO_3_ nanobelts, (b) MnS-incorporated MoO_3_ (MoO_3_/MnS) nanocomposites.

Fig 2 demonstrates HR-TEM images of MoO_3_ and the MoO_3_/MnS nanocomposite, with clearly visible lattice fringes.

The irregular lattice fringes and distorted lattice planes suggest the presence of defect related structural distortion in the nanocomposite [26]. The measured interlayer spacing of MoO_3_ nanobelts was 0.396 nm, which is consistent with previous experimental results. After the incorporation of MnS nanoparticles, the interlayer spacing of the nanocomposite has increased to 0.421 nm. The observed increase in interlayer spacing is primarily due to defect formation induced by MnS incorporation. In addition, local lattice strain and interfacial distortion at the MoO_3_/MnS heterojunctions may also contribute to the lattice expansion. Such combined structural effects facilitate shortened diffusion pathways and may enhance ion accessibility within the electrode material [27]. The increased interlayer distance enhances the material’s stability and reduces charge collapse between layers. Meanwhile, the pores of the nanocomposite accumulate more ions and potentially act as channels for prompt transportation [28]. While any spectroscopic techniques such as XPS, Raman, or EPR could enable quantitative analysis of defect states, the present conclusions are based on qualitative structural and diffraction evidence.

### 3.2 Structural analysis

**Fig 3:**
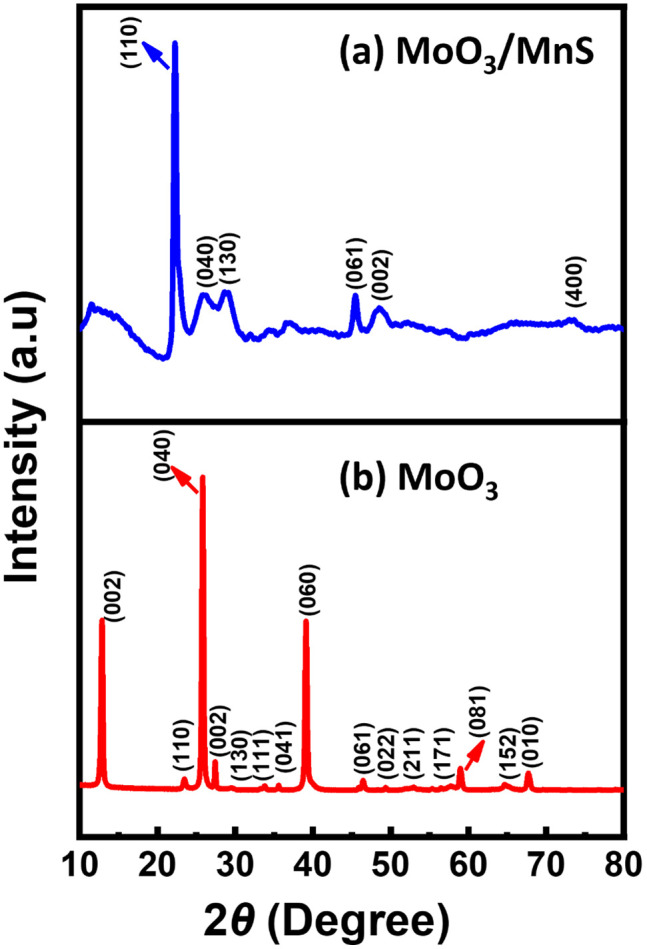
X.ray diffraction pattern of (a) MoO_3_/MnS nanocomposite and (b) MoO_3_.

The crystal structure of MoO_3_ and MoO_3_/MnS was investigated by X-ray diffraction (XRD), as shown in Fig 3.

The diffraction pattern of MoO_3_ in Fig 3(b) displays sharp, well-defined peaks, which are characteristic of a highly crystalline material. The diffraction peaks at 12.7°, 23.3°, 25.7°, 27.4°, and 39.0° correspond to the (020), (110), (040), (021), and (060) planes of orthorhombic (Pbnm space group) MoO_3_ (JCPDS card No. 35–0609), respectively [29]. The prominent peak at 25.7° indicates anisotropic growth along the (004) plane during the synthesis process [30]. The sharp peaks suggest relatively large crystallite sizes. The diffraction peaks of MnS match the JCPDS card number 06–0518, corresponding to cubic α-MnS with a rock-salt structure (S1 Fig). The relatively weak MnS peaks may be attributed to their low weight fraction and uniform dispersion within the MoO_3_ matrix. The XRD pattern of the MoO_3_/MnS nanocomposite in Fig 3(a) shows notable differences. While some of the original peaks of MoO_3_ are retained, additional peaks of MnS appear, confirming the successful formation of MoO_3_/MnS nanocomposite. The characteristic peaks of MnS, at  2θ ≈29∘ and 2θ ≈49∘ can be assigned to the (111) and (220) cubic MnS, respectively. The coexistence of the MoO_3_ and MnS peaks in the nanocomposite’s XRD pattern indicates that the two phases remain separate and that no new phase is forming. In addition, some MoO_3_ peaks are eliminated in the nanocomposite after incorporation of MnS, indicating increased disorder in MoO_3_ [31]. MnS particles embedded within or at the grain boundaries of MoO_3_ may introduce defects that reduce the material’s overall crystalline, leading to missing XRD peaks [32]. The MoO_3_ peaks in the nanocomposite’s XRD pattern are broader and slightly shifted compared to those in the pure MoO_3_ sample. The disappearance and broadening of certain MoO_3_ diffraction peaks in the MoO_3_/MnS nanocomposite may be attributed to reduced crystallite size, defects, and lattice distortion induced by MnS incorporation, as confirmed by HR-TEM (Fig 2). The Scherrer equation for crystallite size estimation was not applied, as its underlying assumptions are not fully valid for this defective nanocomposite system. Moreover, the peak shift to higher angles may be attributed to the introduction of compressive strain in MoO_3_.

Although direct elemental mapping by STEM-EDS was not performed in this study, the incorporation of MnS is supported by XRD, which is also consistent with the morphological transformation observed in TEM, and defect-induced lattice distortion in HR-TEM. Given the low MnS loading (5 wt%), MnS is expected to be finely dispersed within the MoO_3_ matrix.

### 3.3 Electrochemical analysis

**Fig 4:**
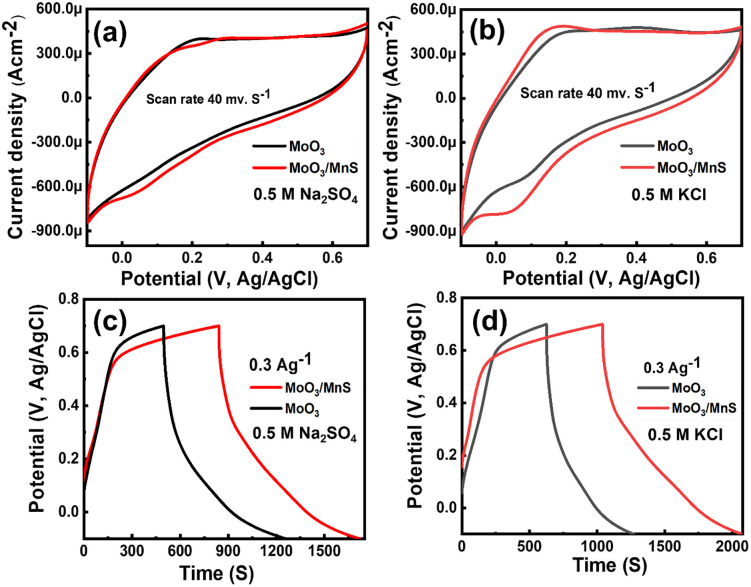
CV curves at 40 mVs^-1^ scan rate for MoO_3_ (a) and MoO_3_/MnS (b); Galvanostatic Charge Discharge (GCD) curves with constant current density 0.3 Ag^-1^ in three three-electrode system of (c) MoO_3_/MnS in Na_2_SO_4_, (d) MoO_3_/MnS in KCl.

Cyclic voltammetry (CV) measurement was performed to investigate the charge storage mechanisms in the synthesized MoO_3_ and MoO_3_/MnS nanocomposite. The CV curves were recorded at a 40 mV s^-1^ scan rate, as shown in Fig 4 (a-b). It is observed that the area enclosed within the CV curves is higher for MoO_3_/MnS nanocomposite than pure MoO_3_ for both 0.5 M Na_2_SO₄, and 0.5 M KCl electrolytes. In case of the 0.5 M Na_2_SO₄, the area for MoO_3_ is $4.22\times 10^{-4}\;A•V•cm^{-2}$, while the MoO_3_/MnS nanocomposite exhibits a larger area of $4.58\times 10^{-4}\;A•V•cm^{-2}$. Similarly, in 0.5 M KCl, the areas are $4.29\times 10^{-4}\;A•V•cm^{-2}$ and $5.08\times 10^{-4}\;A•V•cm^{-2}$ for MoO_3_ and MoO_3_/MnS, respectively. This increase in the enclosed area for the MoO_3_/MnS nanocomposite indicates an enhancement in specific capacitance compared to pure MoO_3_, signifying its superior charge storage capability [30]. The area under the CV curves, is noticeably greater when KCl is used as the electrolyte compared to Na_2_SO₄. This may be due to the smaller ionic radius and higher mobility of K⁺ ions in KCl, which enhance charge storage and ion transport within the electrode structure, as observed in previous studies [33].

For the MoO_3_ sample, the CV curves in both electrolytes exhibit a quasi-rectangular shape, suggesting that its charge storage mechanism is influenced by a combination of double-layer capacitance (EDLC) and pseudo-capacitive processes [34]. In contrast, the MoO_3_/MnS nanocomposite shows a more noticeable deviation from the rectangular shape, especially in the 0.5 M KCl electrolyte. From the CV analysis (i = aνᵇ), the b value is 0.78 that indicates the contributions of both capacitive and diffusion controlled. This indicates the dominant contribution is faradaic pseudo-capacitive charge storage [5]. In the absorption/desorption reaction mechanism, alkali cations (Na^+^ and K^+^) are adsorbed and desorbed into the electrode/electrolyte interface, as illustrated below:


$$\begin{array}{c} {\left({MoO}_{3}\right)}_{surface}+M^{+}+e^{-}↔{\left({MoO}_{3}•M\right)}_{surface} \end{array}$$

$$\begin{array}{c} {\left({MoO}_{3}/MnS\right)}_{surface}+M^{+}+e^{-}↔{\left\{\left({MoO}_{3}/MnS\right)•M\right\}}_{surface} \end{array}$$

Here, M^+^ represents the Na^+^ and K^+^ present in two different electrolytes. In the intercalation mechanism, cations are intercalated into and then extracted from the electrode material. This process involves a reversible structural change, which contributes to the faradaic charge storage.

The intercalation mechanism can be represented as:


$$\begin{array}{c} {MoO}_{3}+M^{+}+e^{-}↔{\left\{\left({MoO}_{3}\right).M\right\}}_{intercalation} \end{array}$$

$$\begin{array}{c} \left({MoO}_{3}/MnS\right)+M^{+}+e^{-}↔{\left\{\left({MoO}_{3}/MnS\right)•M\right\}}_{intercalation} \end{array}$$

The improvement in the specific capacitance of the MoO_3_/MnS nanocomposite can be attributed to several factors. The nanocomposite defective porous morphology with increased surface area, as evident from the SEM images Fig 1(a-b), may facilitate better electrolyte piercing and ion movement, thereby enhancing the charge storage capabilities [35]. Additionally, the incorporation of MnS not only improves electrical conductivity but also provides more active sites for redox reactions, which are crucial for pseudo-capacitance. However, MnS does not serve as the dominant pseudocapacitive phase; rather, it acts as a conductive, defect-inducing component that modifies the MoO_3_ lattice, facilitating enhanced ion diffusion and charge transport.

Fig 4 (c-d) shows the galvanostatic charging curves of MoO_3_ and MoO_3_/MnS electrodes at different electrolytes at a constant 0.3 Ag^-1^ current density. The discharging time of the MoO_3_/MnS is much longer than that of the MoO_3_ electrode [15]. These indicate improvement in charge storage capacity, as shown in the CV curve. The specific capacitance (Cs) from GCD (Galvanostatic Charge Discharge) curves could be determined by applying the following formula$\;{\text{C}}_{\text{s}}=\frac{\text{I}\times \Delta \text{t}}{\Delta \text{V}\times \text{m}}$, where I = discharge current, Δt = discharging time, ΔV = potential window, and m = deposited mass of working electrode. The specific capacitances of MoO_3_ in Na_2_SO_4_ and KCl electrolytes are estimated to be 285 F g^-1^ and 243 F g^-1^, respectively. After inserting the MnS nanoparticles, the MoO_3_/MnS electrode exhibits higher specific capacitance in both electrolytes, and the values are 335 F g^-1^ in Na_2_SO_4_ and 387 F g^-1^ in KCl. The specific capacitance is enhanced due to the porous morphology and the surface defects.

S2b Fig shows the GCD curve of the MoO_3_/MnS electrode in the symmetric two-electrode system, where the specific capacitance is 297 F g^−1^, the energy density is 33.37 W h kg^−1^, and the power density is 450 W kg^−1^. In addition, the nanocomposite exhibits higher capacitance in KCl electrolyte than in Na_2_SO_4_ electrolyte. Generally, K^+^ ions have higher molar conductivity than Na^+^, which easily migrates into the electrode/electrolyte surface [36]. In case of Na_2_SO_4_, the $\overset{\text{2}-}{\underset{\text{4}}{\text{SO}}}$ anion will reduce the mobility of Na^+^ cation, thus making the electrochemical process less effective. Additionally, the K^+^ ions have a lower hydration radius that also helps to increase overall capacitance of the nanocomposite [37].

**Fig 5:**
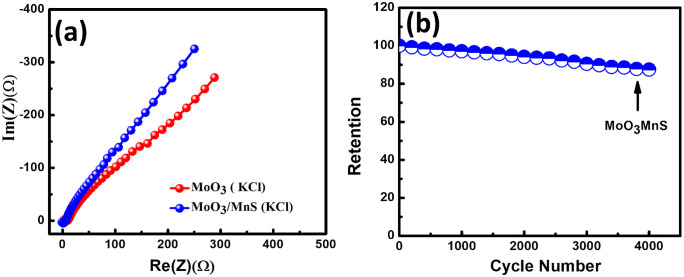
Electrochemical impedance spectra of (a) MoO_3_ and MoO_3_/MnS and (b) Cyclic stability of MoO_3_/MnS nanocomposite in KCl solution.

Electrochemical impedance spectroscopy is another measurement to characterize the electrode materials. From Fig 5(a) exhibits the Nyquist spectra of MoO_3_ and MoO_3_/MnS nanocomposite. In the Warburg region, the MoO_3_/MnS curve is stepper than MoO_3_ which indicates MoO_3_/MnS has low Warburg resistance. The decreased Warburg resistance expedites ion transport and improves capacitive performance.

Cyclic stability is another critical measurement to characterize supercapacitors. Fig 5(b) shows the cyclic stability of MoO_3_/MnS electrode for 4000 charging-discharging cycles. The MoO_3_/MnS electrode exhibits 87% cyclic stability. The enhancement of the nanocomposite’s cyclic stability suggests that the electrode shows a higher specific capacitance as observed for metal sulfide base nanocomposites [38].The common electrochemical activation energy in electrochemistry is the cause of this phenomenon and indicates excellent stability of the novel MoO_3_/MnS.

To summarize, MnS is incorporated into MoO_3_ nanobelts via a two-step hydrothermal method. The hydrothermal process employed in this study is facile and cost-effective. Its high yield and consistent batch reproducibility at the laboratory scale indicate significant potential for future scale-up and industrial application. The diffraction analysis confirmed the successful formation of MoO_3_ nanobelts and MoO_3_/MnS nanocomposite. The incorporated MnS nanoparticles produced defective porous nanofibers and expanded the inter layer spacing. These distinctive features allow ions to move promptly between their interfaces, providing effective charge storage. The MoO_3_/MnS nanocomposite shows 387 F g^-1^ and 335 Fg^-1^ specific capacitances in KCl and Na_2_SO_4_ electrolytes, respectively. The power density of the nanocomposite is 450 W kg^−1^ with an energy density of 33.37 W h kg^−1^.The defective porous structure of MoO_3_/MnS obtained by this simple technique provides an efficient way to produce high quality supercapacitor electrodes for energy storage applications.

## Supporting information

S1 Fig X-ray diffraction pattern of MnS nanoparticles. (DOCX)

S2 Fig CV curve of MoO_3_/MnS (a), and GCD curve of MoO_3_/MnS nanocomposite (b) at two electrode system. (DOCX)

S1 File All relevant data‌‌. (DOCX)
