# Correction: Defect induced improved capacitive performance of MnS incorporated MoO_3_ nanocomposite for supercapacitor electrodes in aqueous electrolytes

**DOI:** 10.1371/journal.pone.0352600

**Published:** 2026-06-24

**Authors:** Mizanur Rahaman, Mehedi Hasan Prince, Saif Mahmud Bijoy, Zakaria Siddiquee, Muhammad Rakibul Islam

The email address for corresponding author Muhammad Rakibul Islam is incorrect. The correct email address is: rakibul@phy.buet.ac.bd.
