# Supplementary material for: Defect induced improved capacitive performance of MnS incorporated MoO3 nanocomposite for supercapacitor electrodes in aqueous electrolytes
Source: PLoS One. 2026 May 18;21(5):e0349019. doi: 10.1371/journal.pone.0349019 (PMC13183187; doi:10.1371/journal.pone.0349019)
Supplement: S1 Fig — (DOCX) [file pone.0349019.s001.docx]

Defect Induced Improved Capacitive Performance of MnS Incorporated MoO_3_ Nanocomposite for Supercapacitor Electrodes in Aqueous Electrolytes

Mizanur Rahaman^1, 2,*,^ Mehedi Hasan Prince^3^, Saif Mahmud Bijoy ^4^, Zakaria Siddiquee^1^, Muhammad Rakibul Islam^2,*^

^1^Department of Physics, Kent State University, Kent, OH 44242, USA

^2^Department of Physics, Bangladesh University of Engineering and Technology, Dhaka, Bangladesh

^3^Department of Materials and Metallurgical Engineering, Bangladesh University of Engineering and Technology, Dhaka, Bangladesh

^4^Advanced Materials and Liquid Crystal Institute, Kent State University, Kent, OH 44242, USA

*Corresponding Author: mrahaman@kent.edu, [rakibul@phy.but.ac.bd](mailto:rakibul@phy.but.ac.bd)

**
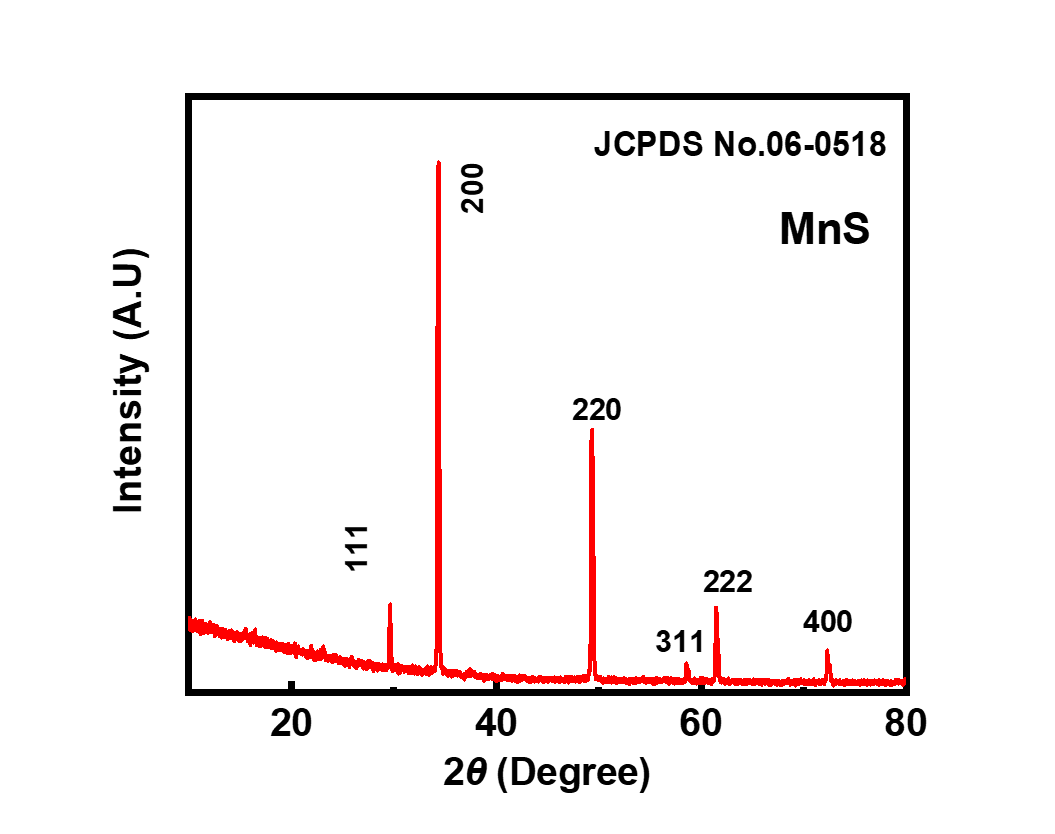
**

**S1 Fig.** X-ray diffraction pattern of MnS nanoparticles
