# Supplementary material for: Defect induced improved capacitive performance of MnS incorporated MoO3 nanocomposite for supercapacitor electrodes in aqueous electrolytes
Source: PLoS One. 2026 May 18;21(5):e0349019. doi: 10.1371/journal.pone.0349019 (PMC13183187; doi:10.1371/journal.pone.0349019)
Supplement: S2 Fig — (DOCX) [file pone.0349019.s002.docx]

Defect Induced Improved Capacitive Performance of MnS Incorporated MoO_3_ Nanocomposite for Supercapacitor Electrodes in Aqueous Electrolytes

Mizanur Rahaman^1, 2,*,^ Mehedi Hasan Prince^3^, Saif Mahmud Bijoy ^4^, Zakaria Siddiquee^1^, Muhammad Rakibul Islam^2,*^

^1^Department of Physics, Kent State University, Kent, OH 44242, USA

^2^Department of Physics, Bangladesh University of Engineering and Technology, Dhaka, Bangladesh

^3^Department of Materials and Metallurgical Engineering, Bangladesh University of Engineering and Technology, Dhaka, Bangladesh

^4^Advanced Materials and Liquid Crystal Institute, Kent State University, Kent, OH 44242, USA

*Corresponding Author: mrahaman@kent.edu, [rakibul@phy.but.ac.bd](mailto:rakibul@phy.but.ac.bd)

**
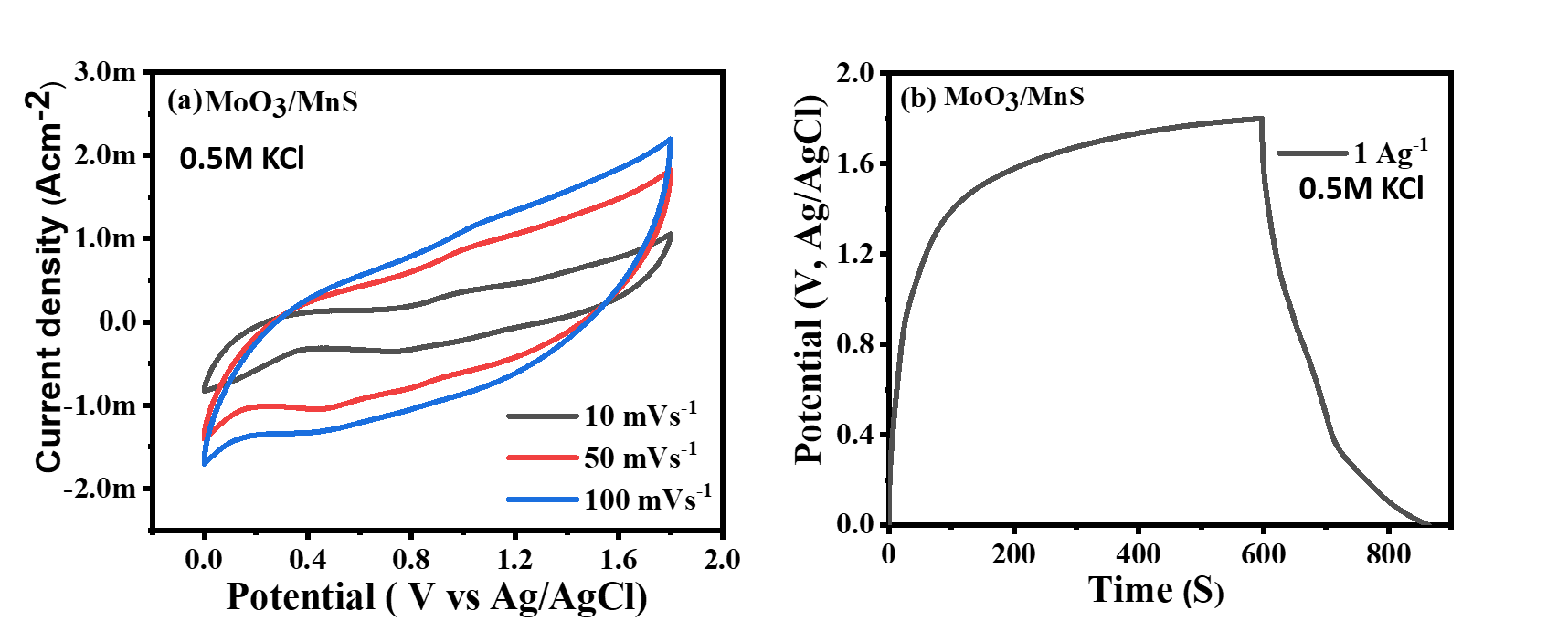
**

**S2 Fig.** CV curve of MoO_3_/MnS (a), and GCD curve of MoO_3_/MnS nanocomposite (b) at two electrode system.

The specific capacitance, energy density and power density of two electrode symmetric system are calculated by using the formulas,

$$C_{s}=\frac{4I\times\Delta t}{\Delta V\times m}$$

$$E_{s}=\frac{Cs\left( \Delta v \right)2\times1000}{8\times3600}$$

$$P_{s}=\frac{E_{s}\times3600}{\Delta T}$$

In the symmetric two electrode system, the MoO_3_/MnS nanocomposite exhibits 297 F g^-1^ specific capacitance at 1 A g^-1^ current density with 33.37 W h kg^−1^ energy density and 450 W kg^−1^ power density.
