# Supplementary material for: Defect induced improved capacitive performance of MnS incorporated MoO3 nanocomposite for supercapacitor electrodes in aqueous electrolytes
Source: PLoS One. 2026 May 18;21(5):e0349019. doi: 10.1371/journal.pone.0349019 (PMC13183187; doi:10.1371/journal.pone.0349019)
Supplement: S1 File — (DOCX) [file pone.0349019.s003.docx]

**Defect Induced Improved Capacitive Performance of MnS Incorporated MoO_3_ Nanocomposite for Supercapacitor Electrodes in Aqueous Electrolytes**

Mizanur Rahaman1, 2,*^,^ Mehedi Hasan Prince3, Saif Mahmud Bijoy4, Zakaria Siddiquee2, Muhammad Rakibul Islam1^,*^

^1^Department of Physics, Bangladesh University of Engineering and Technology, Dhaka, Bangladesh

^2^Department of Physics, Kent State University, Kent, OH 44242, USA

^3^Department of Materials and Metallurgical Engineering, Bangladesh University of Engineering and Technology, Dhaka, Bangladesh

^4^Advanced Materials and Liquid Crystal Institute, Kent State University, Kent, OH 44242, USA

*Corresponding Author: mrahaman@kent.edu, [rakibul@phy.but.ac.bd](mailto:rakibul@phy.but.ac.bd),

**MnS XRD graph**


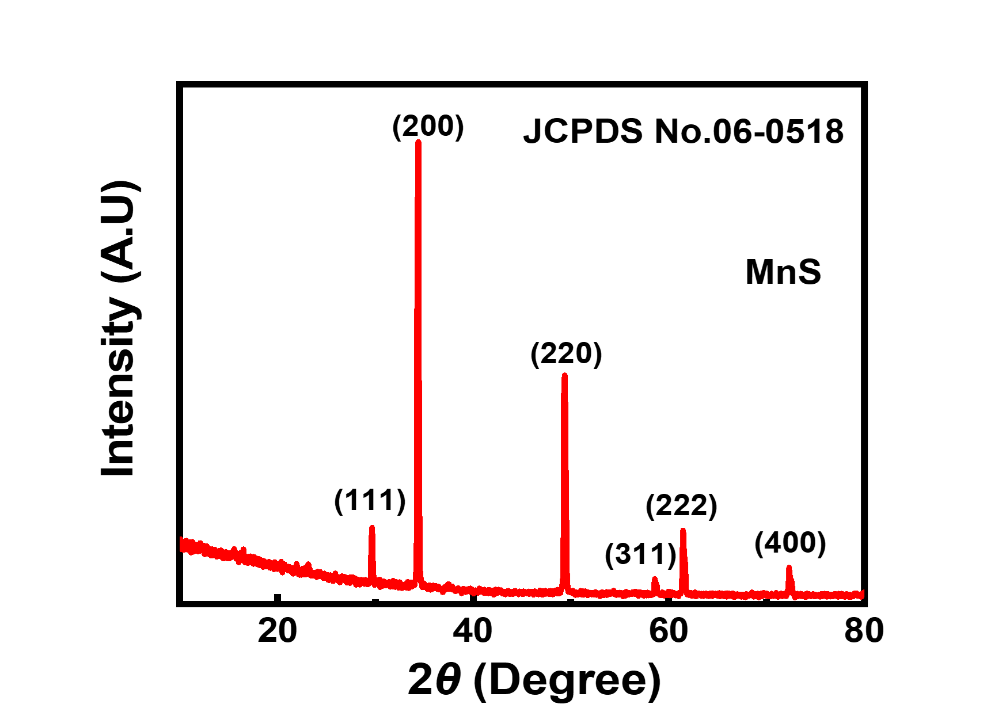


The additional peaks of MnS appear, confirming the successful formation of MoO_3_/MnS nanocomposite. From this graph, the existence of MnS is determined.

**For EIS analysis**

2.65611-3.56037-2.09322---0.85885-3.28872-0.31996-4.83053 3.18618-1.20644 0.91429-4.08977-1.12054-2.9403 0.39067-6.87381 4.32918-0.61522 3.00015 1.6595 1.3457-4.87248 5.99434-1.0086 3.50114-1.20207 3.37448-2.35794 2.63576-4.29238 3.67003-3.963 4.09246-0.0442 6.3301-1.1986 2.32453-1.49324 7.35674-2.52226 5.41542-0.01811 6.25355-1.42909 4.7946-1.13972 7.19096-1.25979 5.5828 0.10279 7.42028-0.59402 4.2147-1.20117 7.26008 0.27263 5.77022 0.36405 7.78086-0.18859 4.38751-1.27256 7.42548-0.11993 5.78623 0.26091 7.07558 0.68059 4.8914-0.1607 7.42723 0.36773 5.88543 0.40752 7.91931 1.04086 4.85191-0.1317 8.20228 0.44314 5.99625 0.38463 8.53875 1.28985 5.15208 0.20728 8.56001 0.91755 6.08832 0.35471 8.89144 1.39795 5.27577 0.19131 8.73213 0.83616 6.1602 0.30195 8.9756 1.23967 5.40957 0.23727 8.8257 0.80751 6.19771 0.28448 9.09697 1.13355 5.4175 0.21008 8.85882 0.70971 6.18175 0.2483 9.19836 1.02705 5.44999 0.23664 8.93012 0.64938 6.23561 0.23094 9.27625 0.94403 5.44816 0.19194 9.03193 0.57569 6.29982 0.23145 9.27752 0.82018 5.45728 0.20195 9.04 0.52999 6.33436 0.19572 9.37131 0.68271 5.48881 0.18668 9.06461 0.44737 6.31996 0.20432 9.39696 0.65929 5.5434 0.22054 9.13204 0.39929 6.32725 0.28191 9.46755 0.57427 5.56809 0.16294 9.10863 0.39409 6.34524 0.23097 9.47205 0.54436 5.54535 0.21498 9.16153 0.41713 6.36887 0.15868 9.48256 0.51348 5.58957 0.23017 9.09978 0.37115 6.3444 0.21364 9.48423 0.41241 5.58346 0.23146 9.17208 0.2982 6.34897 0.19499 9.57205 0.42913 5.58126 0.21051 9.1919 0.34714 6.39726 0.21465 9.55212 0.45794 5.61779 0.2092 9.21609 0.27387 6.40486 0.21386 9.60217 0.39398 5.66388 0.24581 9.17438 0.33436 6.44783 0.25082 9.58267 0.38578 5.68984 0.19782 9.23211 0.38373 6.44549 0.22242 9.63926 0.3841 5.65393 0.283 9.24438 0.35991 6.5034 0.26808 9.6777 0.40257 5.69978 0.257 9.32352 0.33395 6.46567 0.24273 9.66477 0.43307 5.70394 0.29817 9.38773 0.34353 6.4587 0.25201 9.72513 0.41624 5.75963 0.34305 9.38692 0.36618 6.49928 0.35128 9.75493 0.47943 5.77207 0.30587 9.41393 0.43264 6.51795 0.32325 9.80561 0.47394 5.80428 0.35843 9.40081 0.40803 6.563 0.37874 9.78588 0.48316 5.78882 0.37737 9.42263 0.48876 6.58975 0.42602 9.82198 0.55866 5.86692 0.40124 9.51329 0.4577 6.65988 0.47052 9.93499 0.54099 5.91819 0.47592 9.55315 0.47824 6.65202 0.46436 9.98795 0.58271 5.9127 0.48303 9.53201 0.56101 6.68122 0.54804 9.99927 0.66725 5.9332 0.5027 9.56103 0.62426 6.76778 0.57731 10.05 0.72988 5.98993 0.57201 9.57939 0.6337 6.79522 0.60425 10.0851 0.77931 6.06068 0.64213 9.69139 0.7342 6.80646 0.6778 10.1499 0.84637 6.11173 0.6755 9.69491 0.75928 6.83404 0.79081 10.2422 0.87119 6.14463 0.74179 9.77213 0.87729 6.89479 0.83845 10.3137 0.94403 6.20361 0.80861 9.86905 1.02008 6.99056 0.95286 10.4204 1.08511 6.28182 0.87243 9.94952 1.07723 7.04453 1.04931 10.4971 1.17894 6.33717 0.9448 10.0258 1.17353 7.10981 1.16063 10.5894 1.27867 6.41715 1.03741 10.1024 1.28515 7.18506 1.28642 10.6865 1.40698 6.4683 1.13346 10.1864 1.41834 7.27191 1.45284 10.8052 1.54407 6.55477 1.26427 10.2946 1.5892 7.36087 1.6027 10.9049 1.71477 6.63683 1.3871 10.3951 1.74689 7.46196 1.7903 11.0236 1.88599 6.72447 1.53395 10.51 1.93892 7.57469 2.00494 11.1919 2.09192 6.82835 1.69847 10.6619 2.15581 7.68841 2.2282 11.3213 2.33245 6.92015 1.87423 10.7897 2.37946 7.82602 2.49118 11.4704 2.56665 7.02975 2.07739 10.9357 2.64977 7.97532 2.79492 11.639 2.85731 7.15409 2.31256 11.0953 2.9597 8.15214 3.14283 11.8328 3.18495 7.28316 2.59163 11.2847 3.31059 8.34194 3.52204 12.0315 3.5682 7.42085 2.89802 11.479 3.69714 8.56017 3.95418 12.2573 3.997 7.5824 3.25654 11.7053 4.14514 8.79612 4.41704 12.5084 4.48197 7.74796 3.63324 11.9379 4.62576 9.10176 5.00043 12.8091 5.0083 7.9531 4.11389 12.2424 5.22241 9.45156 5.62627 13.1799 5.67546 8.20109 4.64673 12.5979 5.88619 9.67563 6.19166 13.3106 6.40531 8.29932 5.14093 12.7288 6.46647 10.0626 6.9359 13.6798 7.06655 8.5533 5.79635 13.0968 7.26497 10.3584 7.69479 13.9218 7.96542 8.74563 6.48808 13.3473 8.08191 10.9459 8.69279 14.5365 8.91511 9.11372 7.39886 13.9262 9.17975 11.5556 9.75187 15.1606 10.1367 9.5219 8.4082 14.5298 10.3577 12.221 10.9304 15.856 11.4695 9.98908 9.54183 15.1919 11.675 12.8465 12.0116 16.5209 12.9771 10.4158 10.5971 15.8211 12.9012 13.747 13.5369 17.4975 14.3768 11.0582 12.1236 16.7652 14.6327 14.616 14.9882 18.4534 16.3719 11.6887 13.6053 17.6525 16.2908 15.8633 17.0257 19.8835 18.2941 12.6282 15.7055 18.9979 18.6303 17.0383 18.93 21.2947 20.9973 13.6471 17.6651 20.3211 20.8309 18.3814 20.9985 22.8897 23.5739 14.7541 19.8727 21.8523 23.2403 20.0549 23.509 24.9925 26.3687 16.1396 22.5759 23.8028 26.0793 21.7678 26.0868 27.1968 29.781 17.6206 25.3945 25.829 29.0119 23.5959 28.7895 29.5674 33.2756 19.299 28.2923 28.0628 32.0053 25.8298 31.9573 32.5742 36.8601 21.3028 31.7982 30.8126 35.4477 28.4027 35.5468 36.1058 41.1158 23.7564 35.7715 34.0129 39.3112 31.1547 39.289 39.9005 45.8632 26.3931 39.9065 37.4439 43.216 34.1467 43.289 44.1986 50.7236 29.3708 44.3216 41.2291 47.2312 37.6553 47.8634 49.1199 55.8927 32.8518 49.4005 45.5731 51.6807 41.454 52.6897 54.5613 61.6112 36.6892 54.7542 50.2126 56.3143 45.6417 57.9101 60.5782 67.6147 40.9636 60.468 55.2076 61.1085 50.1994 63.46 67.1063 73.8971 45.703 66.5542 60.6428 66.0535 55.486 69.8201 74.7436 80.3458 51.2 73.5467 66.7142 71.5655 61.3071 76.4695 83.0504 87.6199 57.2453 80.8542 73.3491 77.2364 67.012 83.192 91.1493 94.9822 63.296 88.2149 79.4411 82.8011 74.2188 91.8084 101.344 102.088 70.7934 97.5378 87.1865 90.0338 81.4105 99.7939 111.18 111.144 78.3398 106.274 94.4552 96.5177 87.7046 111.193 120.29 118.998 84.9196 118.32 101.12 106.952 97.0005 121.476 132.569 130.877 94.5409 129.706 110.275 115.388 108.157 129.884 146.682 140.407 106.564 139.17 120.712 121.392 119.332 146.305 161.353 146.339 117.987 157.034 132.278 136.256 130.62 158.77 175.024 162.07 130.193 171.111 143.004 146.667 142.637 173.358 189.374 172.536 143.389 187.287 154.54 159.046 155.254 189.245 203.996 184.85 157.447 204.873 166.684 172.694 168.796 207.158 219.367 198.339 172.849 224.507 179.913 187.97 183.177 226.905 235.271 213.536 189.5 245.898 193.927 204.631 198.964 249.446 252.155 230.256 208.038 270.039 209.521 223.583 216.166 275.026 270.085 249.494 228.486 296.782 226.657 244.641 234.428 302.797 288.424 271.102 250.378 325.378 244.608 267.102

Stability data

| 0 | 100 |
| --- | --- |
| 200 | 99.2 |
| 400 | 98.5 |
| 600 | 98.2 |
| 800 | 97.6 |
| 1000 | 97.1 |
| 1200 | 96.6 |
| 1400 | 96.1 |
| 1600 | 95.7 |
| 1800 | 94.9 |
| 2000 | 94.2 |
| 2200 | 93.7 |
| 2400 | 93.4 |
| 2600 | 92.3 |
| 2800 | 91.6 |
| 3000 | 90.5 |
| 3200 | 89.8 |
| 3400 | 88.9 |
| 3600 | 88.6 |
| 3800 | 87.8 |
| 4000 | 87.5 |
