# Supplementary material for: Defect induced improved capacitive performance of MnS incorporated MoO3 nanocomposite for supercapacitor electrodes in aqueous electrolytes
Source: PLoS One. 2026 May 18;21(5):e0349019. doi: 10.1371/journal.pone.0349019 (PMC13183187; doi:10.1371/journal.pone.0349019)
Supplement: Attachment — Submitted filename: Reviewers Comment.pdf [file pone.0349019.s004.pdf]

The manuscript entitled "Defect Induced Improved Capacitive Performance of MnS Incorporated MoO_3 Nanocomposite for Supercapacitor Electrodes in Aqueous Electrolytes" reports the hydrothermal synthesis of a MoO_3/MnS nanocomposite for supercapacitor applications and demonstrates enhanced electrochemical performance compared to pristine MoO_3. While the study is relevant and the results are promising, the following minor issues should be addressed to improve clarity, completeness, and consistency.

1. Electrochemical impedance spectroscopy data are not provided, despite frequent claims of reduced impedance, improved conductivity, and enhanced ion transport. Inclusion of Nyquist plots and fitted equivalent circuit analysis would help support these claims and strengthen the electrochemical discussion.
2. The manuscript repeatedly attributes performance enhancement to increased surface area and porosity; however, no BET surface area measurements are reported. BET analysis is recommended to quantitatively support these claims.
3. The choice of KCl and Na_2SO_4 electrolytes should be more clearly justified, particularly with respect to the electrochemical stability window, ion size and hydration radius, and comparison with more commonly used electrolytes such as KOH or NaOH.
4. The cycling stability is evaluated for only 1000 charge-discharge cycles, which is relatively limited for supercapacitor studies. Recent literature commonly reports stability over 2000-10,000 cycles. The authors should either extend the cycling test or provide a justification for this limitation.
5. Several figure captions lack sufficient detail, such as magnification or scale bars. Figures should be consistently labeled (a, b, c, d) and clearly referenced in the text. Additionally, the phrase "the insect shows" in the SEM description appears to be a typographical error and should be corrected.
6. The manuscript shows inconsistencies in font size and formatting. Some figure captions appear in italics while others do not. The authors should carefully review the manuscript to ensure consistent formatting throughout.
