# Supplementary material for: Defect induced improved capacitive performance of MnS incorporated MoO3 nanocomposite for supercapacitor electrodes in aqueous electrolytes
Source: PLoS One. 2026 May 18;21(5):e0349019. doi: 10.1371/journal.pone.0349019 (PMC13183187; doi:10.1371/journal.pone.0349019)
Supplement: Attachment — Submitted filename: Response of Reviewers.docx [file pone.0349019.s005.docx]

**Reply to the Referees’ comments on Manuscript Number: PONE-D-25-65983**

We thank the referees for the critical review of the manuscript. In the following, we provide our responses to the referee’s comments.

**Reply to the First referees’ comments:**

**Comment (1)** Electrochemical impedance spectroscopy data are not provided, despite frequent claims of reduced impedance, improved conductivity, and enhanced ion transport. Inclusion of Nyquist plots and fitted equivalent circuit analysis would help support these claims and strengthen the electrochemical discussion.

**Response 1:** We thank the referee for his/her thoughtful comment. The electrochemical impedance spectra data are provided and explain why ion transportation in increased in the nanocomposite. The new lines are added in the revised manuscript “Electrochemical impedance spectroscopy is another measurement to characterize the electrode materials. From Fig. 5(a) exhibits the Nyquist spectra of MoO_3_ and MoO_3_/MnS nanocomposite. In the Warburg region, the MoO_3_/MnS curve is stepper than MoO_3_ which indicates MoO_3_/MnS has low Warburg resistance. The decreased Warburg resistance expedites ion transport and improves capacitive performance.”

**Comment (2)** The manuscript repeatedly attributes performance enhancement to increased surface area and porosity; however, no BET surface area measurements are reported. BET analysis is recommended to quantitatively support these claims

**Response 2:** We thank the referee for his/her thoughtful comment. The BET analysis is very helpful to understand the surface area and porosity, but this characterization technique is not available in Bangladesh and Kent State University.

**Comment (3)** The choice of KCl and Na_2_SO_4_ electrolytes should be more clearly justified, particularly with respect to the electrochemical stability window, ion size and hydration radius, and comparison with more commonly used electrolytes such as KOH or NaOH.

**Response 3:** We thank the referee for his/her thoughtful comment. to the electrochemical stability window is around 1.24 V and K⁺ (ionic radius 138 pm) vs. Na⁺ (102 pm). The hydration radius is (3.31 Å) for K⁺ and (3.58 Å) for Na⁺. On the other hand, the KOH or NaOH has large ionic radius that reduce the electrochemical performance.

**Comment (4)** The cycling stability is evaluated for only 1000 charge–discharge cycles, which is relatively limited for supercapacitor studies. Recent literature commonly reports stability over 2000-10,000 cycles. The authors should either extend the cycling test or provide a justification for this limitation.

**Response 4:** We thank the referee for the comment. The electrochemical stability of the nanocomposite tested again up to 4000 charge–discharge cycles. In the revised manuscript, the latest portion is “Cyclic stability is another critical measurement to characterize supercapacitors. Figure 5(a) shows the cyclic stability of MoO_3_/MnS electrode for 4000 charging-discharging cycles. The MoO_3_/MnS electrode exhibits 87% cyclic stability. The enhancement of the nanocomposite’s cyclic stability suggests that the electrode shows a higher specific capacitance as observed for metal sulfide base nanocomposites34.The common electrochemical activation energy in electrochemistry is the cause of this phenomenon and indicates excellent stability of the novel MoO_3_/MnS.”

**Comment (5)** Several figure captions lack sufficient detail, such as magnification or scale bars. Figures should be consistently labeled (a, b, c, d) and clearly referenced in the text. Additionally, the phrase “the insect shows” in the SEM description appears to be a typographical error and should be corrected.

**Response 5:** We thank the referee for the comment. The magnification or scale bars have already been included, and all the figures are labeled earlier. The typographical error is corrected as Inset.

**Comment (6)** The manuscript shows inconsistencies in font size and formatting. Some figure captions appear in italics while others do not. The authors should carefully review the manuscript to ensure consistent formatting throughout.

**Response 6:** We thank the reviewer for pointing out these formatting inconsistencies. The manuscript has now been carefully reviewed and revised to ensure uniform font size, formatting, and figure caption style throughout the document. We believe the updated manuscript presents a consistent and professional appearance.

**Reply to the Second referees’ comments:**

**Comment (1)** The manuscript discusses MnS incorporation effects extensively; however, no standalone FESEM image of pristine MnS nanoparticles is provided, while FESEM images are shown only for MoO₃ and MoO₃/MnS. This omission makes it difficult to confirm MnS size, dispersion, and morphology prior to composite formation.

**Response 1:** We thank the referee for the comment. The FESEM image of MnS is included in the inset of Fig. 1(a). The FESEM image of MnS is included here,

**Comment (2)** The authors claim that MnS “shrinks the MoO₃ nanobelts into nanofibers,” yet no quantitative statistical analysis (diameter distribution, fiber length histograms) is provided to support this transformation.

**Response 2:** We thank the referee for the comment. The new line is added in the latest manuscript. The new line is “By using ImageJ software, the measured nanofibers diameter is 30-40 nm.”

**Comment (3)** The successful incorporation and spatial distribution of MnS within the MoO₃ matrix are not convincingly demonstrated. STEM-EDS elemental mapping is essential to confirm homogeneous Mn and S distribution.

**Response 3:** We thank the reviewer for this valuable suggestion. We agree that STEM-EDS elemental mapping would provide direct confirmation of the spatial distribution of Mn and S within the MoO_3_ matrix.

In the present work, STEM-EDS analysis was not included, as the primary focus was on correlating structural modification and electrochemical performance. The incorporation of MnS was supported through XRD phase identification, TEM/HR-TEM morphology analysis, and consistent electrochemical enhancement. Given the low MnS content (5 wt%) and its role as a structure-modifying additive rather than a primary electroactive phase, these techniques were considered sufficient for the scope of this study.

We acknowledge that STEM-EDS mapping would provide additional insight into elemental homogeneity, and we have now explicitly mentioned this as a limitation of the current work. In future studies, we will incorporate advanced elemental-mapping techniques to further indicate the nanoscale distribution of MnS within the MoO_3_ framework. The updated portion is “Although direct elemental mapping by STEM-EDS was not performed in this study, the incorporation of MnS is supported by XRD, which is also consistent with the morphological transformation observed in TEM, and defect-induced lattice distortion in HR-TEM. Given the low MnS loading (5 wt%), MnS is expected to be finely dispersed within the MoO_3_ matrix.”

**Comment (4)** Defects are inferred primarily from HRTEM lattice distortion. However, no complementary techniques such as XPS (Mo⁶⁺/Mo⁵⁺ ratio), Raman defect analysis, or EPR are used to quantitatively validate defect density.

**Response 4:** We thank the reviewer for this constructive comment. We agree that any spectroscopic techniques, such as XPS, Raman spectroscopy, and EPR, could provide valuable quantitative information on defect chemistry and oxidation-state variations.

In the present study, defect formation was understood qualitatively, not quantitatively, based on multiple indirect observations, including lattice distortion and irregular fringes in HR-TEM images, suppression and broadening of MoO_3_ diffraction peaks in XRD, and increased interlayer spacing. These combined structural features are widely used to understand structural defects.

We acknowledge that the absence of any spectroscopic defect quantification experiment represents a limitation of the current work. To avoid overinterpretation, we have revised the manuscript slightly to clearly state that defect-related conclusions are based on qualitative indirect understating rather than exact quantitative defect density measurements. Future studies will incorporate advanced spectroscopic techniques to further quantify defect states and correlate them with electrochemical performance.

**Comment (5)** The reported increase from 0.396 nm to 0.421 nm is attributed to defect generation, but alternative causes such as strain, intercalation, or phase distortion are not discussed or ruled out.

**Response 5:** We thank the reviewer for this insightful comment. We agree that interlayer expansion in layered metal oxides can originate from multiple factors, including defect formation, lattice strain, interfacial distortion, and possible ion accommodation, as the reviewer mentioned.

In the present manuscript, the increase in interlayer spacing in the MoO_3_/MnS nanocomposite was primarily attributed to defect generation and lattice distortion induced by MnS incorporation. However, we acknowledge that local strain and interfacial effects at the MoO_3_–MnS heterointerfaces may also contribute to the observed interlayer expansion. We have revised the manuscript to clarify that the interlayer expansion may result from the combined effects of defect formation, strain, and interfacial lattice distortion, rather than defect generation alone. We hope this clarification provides a more comprehensive and balanced interpretation of the structural data.

**Comment (6)** MnS polymorphism strongly influences electrochemical behavior, yet the manuscript does not clarify which MnS phase (α, β, γ) is present, nor its electrochemical relevance.

**Response 6:** The MnS phase was identified based on XRD analysis (JCPDS card No. 06-0518), which corresponds to cubic α-MnS (rock-salt structure). The characteristic diffraction peaks of MnS observed in the nanocomposite confirm the presence of this phase. However, due to the lower MnS content (5 wt%) and its incorporation within the MoO_3_ matrix, the MnS peaks appear relatively weak.

One important thing about this study is that MnS does not function as the primary pseudocapacitive material. Instead, α-MnS acts as a conductive, structure-modifying entity, inducing defects and lattice distortion, thereby enhancing ion transport and electrochemical accessibility. Therefore, the improved electrochemical performance of the MoO_3_/MnS nanocomposite is primarily driven by defect-enhanced MoO_3_ charge storage, with MnS contributing indirectly through enhanced conductivity and defect engineering.

To clarify this point, we have revised the manuscript to explicitly identify the MnS polymorph and to better describe its electrochemical role within the composite system.

**Comment (7)** The authors attribute missing MoO₃ peaks to disorder induced by MnS incorporation, but crystallite size estimation (Scherrer analysis) or Williamson–Hall strain analysis is absent.

**Response 7:** We thank the reviewer for this thoughtful comment. We agree that peak broadening and suppression in XRD patterns can arise from multiple factors, including reduced crystallite size, lattice strain, and structural disorder.

In the work, the MoO_3_/MnS nanocomposite showed a highly defective and anisotropic morphology, as indicated in the FE-SEM, TEM, and HR-TEM analyses. The incorporation of MnS induces significant lattice distortion and defect formation, all of which contribute to peak broadening and the disappearance of certain diffraction peaks. Under such conditions, applying the Scherrer equation may yield misleading results, as it assumes strain-free, isotropic crystallites (which is not the case here).

Therefore, we relied on direct microscopic images (TEM and HR-TEM) to support our argument for structural disorder and reduced crystallinity, rather than applying Scherrer or Williamson-Hall analyses. To avoid ambiguity, we have revised the manuscript to clarify this point and mentioned that the missing peaks are primarily associated with defect-induced disorder and strain rather than crystallite-size reduction alone.

**Comment (8)** The enhancement in capacitance is repeatedly attributed to increased surface area, but no BET or pore-size distribution data are provided to support this central claim.

**Response 8:** We thank the referee for his/her thoughtful comment. The BET analysis is very helpful to understand the surface area and porosity, but this characterization technique is not available in Bangladesh and Kent State University. From the FESEM image, the morphology is converted from nanobelts to nanofibers. This increases surface area relative to volume ratio. The example is,

**Comment (9)** The CV analysis lacks b-value determination (i = aνᵇ) to differentiate capacitive vs diffusion-controlled contributions, which is critical for pseudocapacitive systems.

**Response 9:** We thank the referee for his/her thoughtful comment. In the CV analysis, the b value is 0.78 which indicates the mixed state. In the revised manuscript, the new line is added. The new line is” From the CV analysis (i = aνᵇ), the b value is 0.78 that indicates the contributions of both capacitive and diffusion controlled.”

**Comment (10)** Claims of reduced impedance and improved charge transport are made without Nyquist plots or fitted equivalent circuit models.

**Response 10:** We thank the referee for his/her thoughtful comment. The electrochemical impedance spectra data are provided and explain why ion transportation in increased in the nanocomposite. The new lines are added in the revised manuscript “Electrochemical impedance spectroscopy is another measurement to characterize the electrode materials. From Fig. 5(a) exhibits the Nyquist spectra of MoO_3_ and MoO_3_/MnS nanocomposite. In the Warburg region, the MoO_3_/MnS curve is stepper than MoO_3_ which indicates MoO_3_/MnS has low Warburg resistance. The decreased Warburg resistance expedites ion transport and improves capacitive performance.”

**Comment (11)** The manuscript does not clearly report active material mass loading per electrode, which is essential for benchmarking specific capacitance values.

**Response 11:** We thank the referee for his/her thoughtful comment. The active material mass per electrode is 0.3 mg. In the characterization section the new lines are added. The new lines are “For the working electrodes, polyvinyl alcohol (C2H4O) and dimethyl sulfoxide (C2H6OS) solvents were mixed with the electrode materials. Afterwards, these mixed solutions were put uniformly (0.3 mg active mass loading) via drop casting on the surface of modified graphite electrode and dried 30 min at 60 °C.”

**Comment (12)** Stability is evaluated only up to 1000 cycles, which is significantly lower than contemporary supercapacitor standards (≥5000–10000 cycles).

**Response 12:** We thank the referee for his/her thoughtful comment. The electrochemical stability of the nanocomposite tested again up to 4000 charge–discharge cycles. The modified lines are “Cyclic stability is another critical measurement to characterize supercapacitors. Figure 5(a) shows the cyclic stability of MoO_3_/MnS electrode for 4000 charging-discharging cycles. The MoO_3_/MnS electrode exhibits 87% cyclic stability. The enhancement of the nanocomposite’s cyclic stability suggests that the electrode shows a higher specific capacitance as observed for metal sulfide base nanocomposites34.The common electrochemical activation energy in electrochemistry is the cause of this phenomenon and indicates excellent stability of the novel MoO_3_/MnS.”

**Comment (13)** The symmetric device performance is reported, but no Ragone plot comparison with recent MoO₃-based devices is provided.

**Response 13:** We thank the referee for his/her thoughtful comment. The Ragone plot is very effective to compare the device performance. In this work, we focused on the three-electrode performance which gave the electrode materials performance. We obviously include Ragone plot and compare when we will work on device performance and their comparison.

**Comment (14)** The explanation for superior KCl performance is qualitative. Ion diffusion coefficients or EIS-based electrolyte resistance comparisons are not presented.

**Response 14:** We thank the referee for his/her thoughtful comment. The EIS graph is provided and explains why resistance is decreased in the nanocomposite. In this paper, we found why MoO₃/MnS nanocomposite shows higher specific capacitance in both electrolytes. In our manuscript, we also explain why KCl is better. We are working on electrolyte-based supercapacitor where we are trying to find out about EIS-based electrolyte resistance.

**Comment (15)** The Introduction lacks a systematic comparison with recent MoO₃/MnS, MoO₃/MoS₂, MoO₃/metal sulfide, and defect-engineered MoO₃ composites reported in the last 5 years.

**Response 15:** We thank the referee for his/her thoughtful comment. The Introduction has been revised to include a systematic discussion of recent MoO_3_-based composites, including MoO_3_/MoS_2_, MoO_3_/metal sulfide, and defect-engineered MoO_3_ systems reported in the last five years.

**Comment (16)** A benchmark table comparing specific capacitance, energy density, cycling life, and synthesis method with similar MoO₃-based composites is missing.

**Response 16:** We thank the referee for his/her thoughtful comment. In the introduction section, the Mo base previous has already included specific capacitance, cyclic stability, synthesis method etc.

**Comment (17)** The statement “no prior work has been done” is too strong. Several MoO₃–sulfide hybrid systems exist, and the novelty should be reframed more cautiously.

**Response 17:** We have revised the manuscript to use more cautious and precise language, clearly positioning our work within the existing literature and highlighting its specific novelty. This revision improves accuracy and better aligns with current research.

**Comment (18)** The manuscript does not separate MnS redox contribution vs MoO₃ intercalation contribution, nor does it provide supporting redox peak analysis.

**Response 18:** We thank the referee for his/her thoughtful comment. As far as we know, the redox peak analysis is included in the battery related paper. For your suggestion, we may include redox contribution and intercalation contribution in our upcoming manuscript.

**Comment (19)** Although hydrothermal synthesis is claimed to be scalable, no yield, batch reproducibility, or scale-up discussion is included.

**Response 19:** In this work, we claimed the scalability based on the facile, low-temperature, solution-based nature of the hydrothermal process, which has been previously reported as scalable in the literature. Although a full industrial-scale demonstration is beyond the scope of the present study. However, to address the reviewer’s concern, we have revised the manuscript to (i) moderate the wording related to scalability and (ii) include a brief discussion on yield, reproducibility, and scale-up potential in the introduction section.

**Comment (20)** Several sections contain grammatical errors, repetitive explanations, and unclear phrasing that obscure scientific meaning, requiring professional language editing.

**Response 20:** The manuscript has now been thoroughly revised to improve grammar, sentence structure, and overall readability. Redundant explanations have been removed, and unclear phrasing has been rewritten to express the exact scientific meaning. We believe the revised manuscript now meets the standards of professional scientific writing and is significantly clearer.
